# Prevalence, characteristics and risk factors of birth defects in central China livebirths, 2015–2022

**DOI:** 10.3389/fpubh.2024.1341378

**Published:** 2024-09-18

**Authors:** Ping Luo, Qian Li, Bin Yan, Yusha Xiong, Ting Li, Xiao Ding, Bing Mei

**Affiliations:** ^1^Department of Laboratory Medicine, Jingzhou Hospital Affiliated to Yangtze University, Jingzhou, China; ^2^Department of Laboratory Medicine, Gongan County Maternal and Child Health Care Hospital, Jingzhou, China

**Keywords:** birth defects, prevalence, risk factors, characteristics, epidemiology, congenital malformations, weight, age

## Abstract

**Objective:**

This study analyzed the prevalence, epidemiological characteristics and risk factors of birth defects among livebirths in central China, aiming to provide evidences for the prevention of birth defects and government Decision-makings.

**Methods:**

Birth data from China’s Hubei Province between 2015 and 2022 were collected, including basic information of the livebirths, the mothers and the fathers, as well as information about delivery and each prenatal examination. The livebirths prevalence of birth defects was calculated and the trends were mapped. The basic characteristics of birth defects were evaluated by the difference analysis between case and health groups. Univariate and multivariate Poisson regression was performed to examine the independent risk factors for birth defects.

**Results:**

Among 43,568 livebirths, 166 livebirths were born with birth defects, resulted in a total prevalence rate of 3.81 per 1,000 livebirths, showing a remarkable uptrend from 0.41per 1,000 livebirths in 2015 to 9.23 per 1,000 livebirths in 2022. The peak of the prevalence was in January and February. Congenital malformation of the musculoskeletal system was the main type of birth defect in central China livebirths, followed by cleft lip and cleft palate. Overall, newborns with birth defect had significantly earlier delivery gestational age, poorer health and higher proportion of infants with low birth weight than healthy births. The gender of livebirths, excess weight at delivery (≥80 kg) of mothers, more than 2 times of gravidity or parity of mothers, and advanced paternal age (≥40 years) were independent risk factors for birth defects (or specific birth defects).

**Conclusion:**

The livebirths prevalence of birth defects shows increasing trend in central China, which deserves the attention of the government and would-be parents. Elevated paternal age, excess maternal weight, gravidity and parity should be considered when planning their families.

## 1 Introduction

Birth defect is a worldwide public health problem, which brings misery to children and families and increases financial burden to the country and society (1, 2). Researchers and clinicians worldwide are trying their best to find risk factors for birth defects and are constantly improving prenatal screening and diagnostic techniques to minimize the prevalence. However, the prevalence of birth defect in China remains stubbornly high (3, 4), despite the efforts of the government, such as free premarital medical examination, free distribution of folic acid before and during pregnancy, free prenatal testing for chromosomal abnormalities and infectious diseases transmitted vertically from mother to child, continuous improvement of perinatal education and antenatal care services.

Although there have been a large number of epidemiological studies on birth defects, results regarding the prevalence and risk factors were inconsistent in different countries, even in different regions of the same country, due to differences in race, culture, living environment and government policies (3–6). The prevalence and types of birth defects have changed over time, too (4, 6). At present, investigations of birth defects in China have mainly focused on the provinces of Guangxi (7), Hunan (4, 8), Zhejiang (9) and Jiangsu (3), while fewer data in central China have been reported, especially in recent years. In this study, we analyzed the trends of prevalence and main types of birth defects among livebirths from 2015 to 2022 in central China, and further explored the risk factors, aiming to provide evidences for the prevention of birth defects and government Decision-making.

## 2 Methods

### 2.1 Data collection

The data used for analysis in this study was from the database of “HuBei Province Maternal and Children’s Health Services Information Management System” which recorded sociodemographic data of the fathers and mothers, information on each prenatal examination, and birth information of newborns born in Hubei Province. All hospitals in Hubei Province that offered service of prenatal examination and delivery care were required by policy to register and upload the above information into the system.

Every pregnant woman was provided Maternal and Child Health Handbook at the first prenatal examination, which was issued by the National Health Commission of China, used countrywide and integrated pregnancy health care, hospital delivery, child health care, child vaccination and family planning services. The handbook detailed the precautions during pregnancy, items and gestational weeks for prenatal examinations, which was personalized in different provinces according to local prevalence and economic conditions. For prenatal screening in Hubei Province, in addition to routine examinations and nutritional guidance for each prenatal examination, it was a routine screening for infectious diseases, thalassemia, fasting blood glucose and nuchal translucency (NT, a technique for detecting fetal chromosomal abnormalities by ultrasound) before 13 weeks of gestation, Down’s screening or Non-invasive Prenatal Testing (NIPT) at 14–20 gestational weeks, four-dimensional color Doppler ultrasound at 20–24 gestational weeks screening for congenital heart disease and other fetal malformations, and 75 g oral glucose tolerance test (OGTT) at 24–28 gestational weeks.

Birth defects were diagnosed by physical examination, ultrasonography, X-ray examination, and/or genetic diagnostic methods, based on the Chinese National Criteria of Birth Defects and Tiny Deformities and the clinical modification codes as congenital malformations, deformations, and chromosomal abnormalities (codes Q00–Q99) of the International Classification of Diseases, 10th Revision (ICD-10) (7). Trained clinicians from each registered hospital were responsible for the diagnostic confirmation and uploading it into the system.

Data of newborns who born in Hubei Province but did prenatal examinations outside Hubei Province, were not included in this study because these data were not uploaded to the database. This study only analyzed birth defects diagnosed in perinatal period. Fetuses aborted, induced and stillborn due to birth defects were excluded from our study.

Due to the heavy workload of data collation, verification and analysis, we downloaded all birth data of livebirths born in Gongan County, Jingzhou City, Hubei Province between January 2015 and May 2022 from the above system for analysis, given that the corresponding uploaded data in this area was relatively complete.

### 2.2 Categories of birth defect diagnoses in newborns

According to the 10th Revision of International Classification of Diseases, the specific codes for congenital malformations, deformations, and chromosomal abnormalities were Q00–Q99, which were further subdivided into Q00–Q07 (congenital malformations of the nervous system), Q10–Q18 (congenital malformations of eye, ear, face and neck), Q20–Q28 (congenital malformations of the circulatory system), Q30–Q34 (congenital malformations of the respiratory system), Q35–Q37 (cleft lip and cleft palate), Q38–Q45 (other congenital malformations of the digestive system), Q50–Q56 (congenital malformations of genital organs), Q60–Q64 (congenital malformations of the urinary system), Q65–Q79 (congenital malformations and deformations of the musculoskeletal system), Q80–Q89 (other congenital malformations), Q90–Q99 (chromosomal abnormalities). Infants with more than one defect category were included in each applicable major defect category.

### 2.3 Definition

Low birth weight infant: newborns with a birth weight of less than 2,500 g (10). Premature infant: infants up to 28 weeks gestation age but less than 37 weeks (10). Health status of newborns: infants with no complications at birth are assessed as good; infants with non-life-threatening complications are assessed as average; and infants with life-threatening complications are assessed as poor (11). Weight gained: the amount of weight a pregnant woman gains from the beginning of pregnancy to the moment of delivery (12). Abnormal pregnancy-labor history: history of miscarriage, induction, premature delivery, dystocia, stillbirth, birth defects, neonatal death, hydatidiform mole and ectopic pregnancy. High-risk pregnancy: pregnancy with risk factors as follows: (1) special basic condition of the mother (age < 18 years or age ≥ 35 years, weight ≤ 40 kg or weight > 80 kg, height ≤ 1.45 m, thoracic deformity, birth canal deformity and narrow pelvis); (2) history of abnormal pregnancy and childbirth (abortion ≥2 times, spontaneous abortion ≥3 times, preterm birth ≥2 times, years of infertility, history of stillbirth or neonatal death, history of vaginal dystocia, history of postpartum hemorrhage, history of oaf, history of neonatal hemolysis, pregnancy after fallopian tube anastomosis); (3) virus infection, occupational toxicant exposure, smoking or taking contraindicated drugs for pregnant women, and exposure to radioactivity; (4) pregnancy coexisted diseases (pregnancy complicated with heart disease, diabetes, hypertension, kidney disease, liver disease, tuberculosis, hyperthyroidism or hypothyroidism, hematologic disease, anemia, tumor, etc.); (5) pregnancy complication (gestational hypertension, prenatal hemorrhage, fetal malposition, threatened preterm birth, overdue pregnancy, abnormal amniotic fluid volume, twin or macrosomia, fetal intrauterine growth retardation, mother–child blood incompatibility, premature rupture of membranes, etc.); (6) factors of social environment and psychology (financial difficulties, poor transportation, alcoholism, anxiety, fear, mental disorders, depression, etc.). Exposure to suspected teratogens in the first trimester of pregnancy: virus infection, occupational toxicant exposure, smoking or taking contraindicated drugs for pregnant women, and exposure to radioactivity. The above three definitions (abnormal pregnancy-labor history, high-risk pregnancy, exposure to suspected teratogens in the first trimester of pregnancy) were from the China’s Guideline of preconception and prenatal care (2018) (13).

### 2.4 Ethical approval

Ethics approvals were obtained by the ethics committees of Jingzhou Hospital Affiliated to Yangtze University (number: 2022–049-01) and Gongan County Maternal and Child Health Care Hospital (number: 2022-02-01). This study followed the principles of the Declaration of Helsinki. Informed consent was waived by the committees.

### 2.5 Statistical analysis

The prevalence of birth defects was calculated using the method recommended by the European Surveillance of Congenital Anomalies (EUROCAT), which was calculated as the total number of livebirths with birth defects divided by the total number of livebirths (7, 14). Normality of distribution for continuous variables was tested by the Kolmogorov–Smirnov test. The basic characteristics of birth defects were evaluated by the difference analysis between case and control groups. Two-sample t test for continuous variables, and Chi-square test, Fisher’s exact test or Mann–Whitney *U* test for categorical variables were used to evaluate the difference in means and proportions between case and health groups. The Contingency coefficient *C* was calculated to evaluate the correlation and closeness degree when the difference in constituent ratio between the two groups was statistically significant. Univariate and multivariate Poisson regression was performed to examine the independent risk factors for birth defects. For this study, *p* < 0.05 was accepted as statistically significant. Analyses were performed with SPSS 25.0 software.

## 3 Results

### 3.1 Prevalence and trends of birth defects

**Figure 1:**
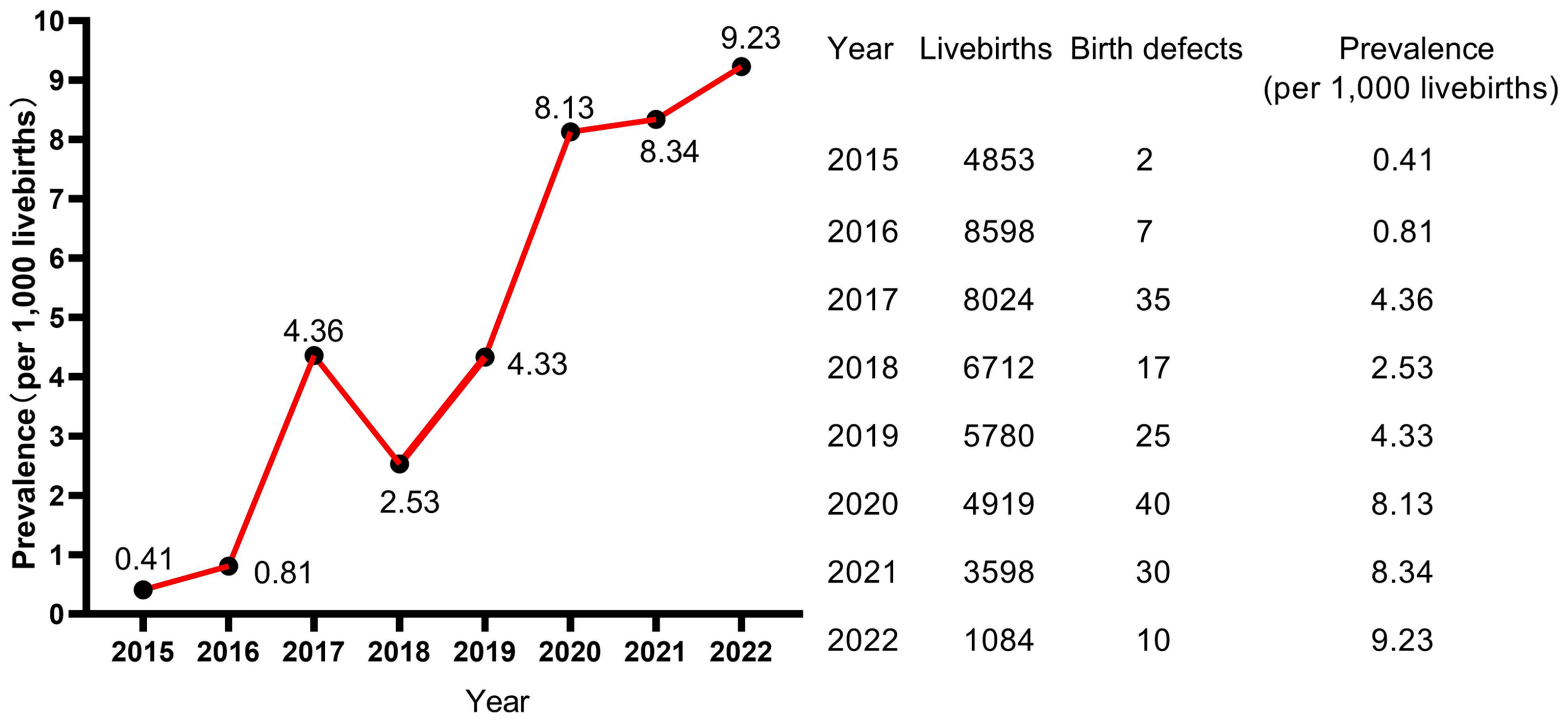
Prevalence and trend of birth defects.

**Figure 2:**
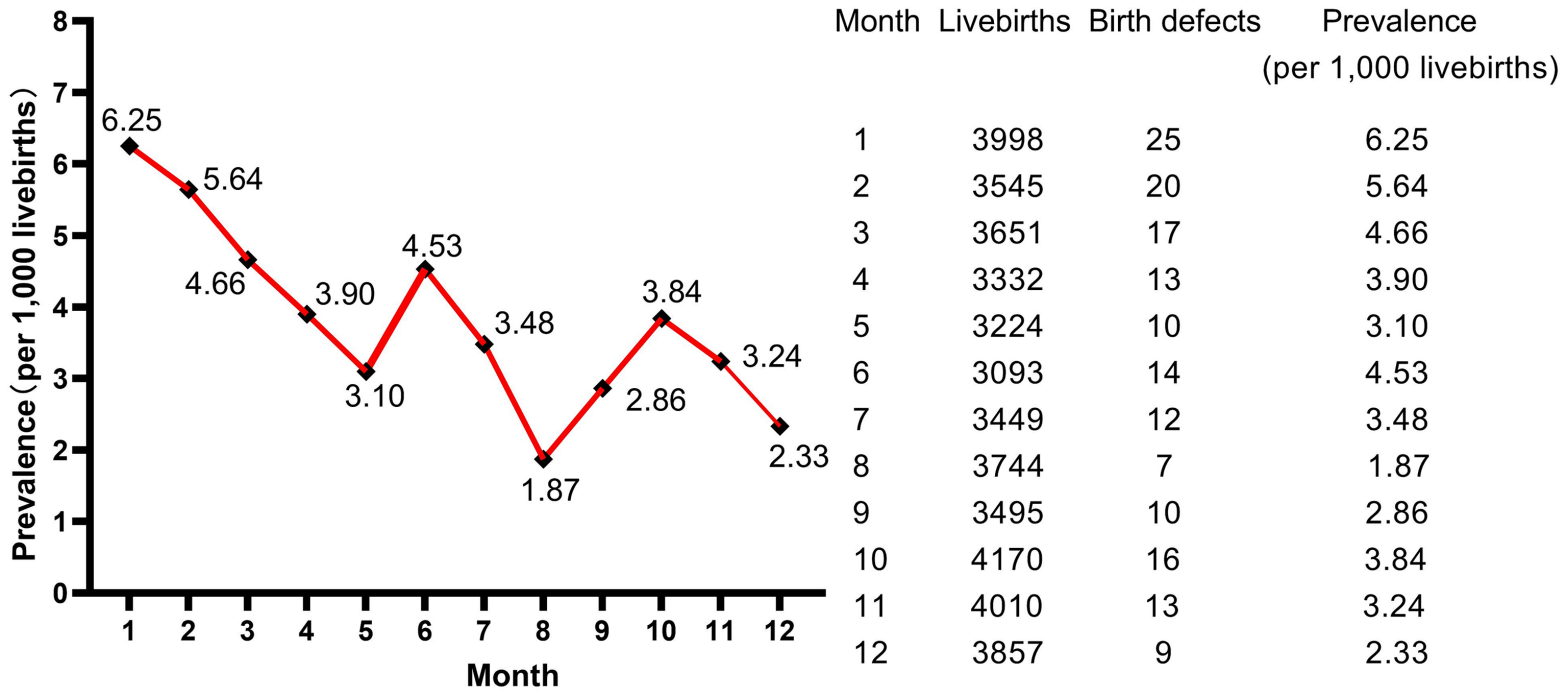
Monthly trend of birth defects.

A total of 43,568 livebirths were included in this study. Among them, 166 newborns were born with birth defects, giving a total prevalence rate of 3.81 per 1,000 livebirths. As shown in Figure 1, the livebirths prevalence of birth defects increased steadily every year, and the difference of prevalence was statistically significant (*p* < 0.001, not shown). Moreover, the peak of the prevalence was in January and February, meaning that newborns born in January and February have a higher prevalence of birth defects (Figure 2).

**Figure 3:**
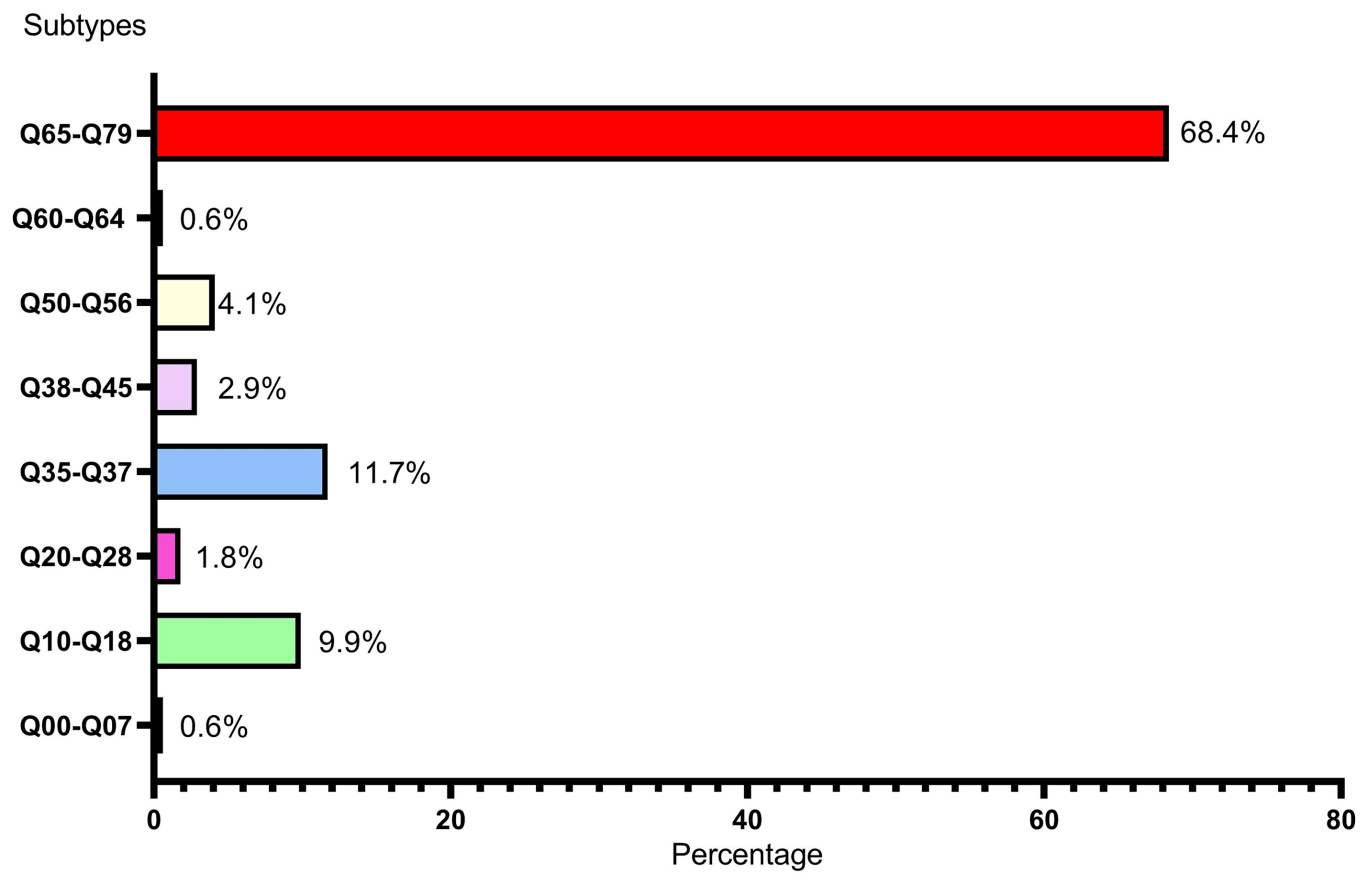
Subtypes and percentages of birth defects Q00–Q07: congenital malformations of the nervous system, Q10–Q18: congenital malformations of eye, ear, face and neck, Q20–Q28: congenital malformations of the circulatory system, Q35–Q37: cleft lip and cleft palate, Q38–Q45: other congenital malformations of the digestive system, Q50–Q56: congenital malformations of genital organs, Q60–Q64: congenital malformations of the urinary system, Q65–Q79: congenital malformations and deformations of the musculoskeletal system.

In this study, among the 166 livebirths with birth defects, 163 newborns were single-system birth defects (98.19%), and only 3 newborns were combined with multi-system birth defects (1.81%). A total of eight types of birth defects were observed in our study population, including congenital malformations of the nervous system (Q00–Q07), congenital malformations of eye, ear, face and neck (Q10–Q18), congenital malformations of the circulatory system (Q20–Q28), cleft lip and cleft palate (Q35–Q37), other congenital malformations of the digestive system (Q38–Q45), congenital malformations of genital organs (Q50–Q56), congenital malformations of the urinary system (Q60–Q64), congenital malformations and deformations of the musculoskeletal system (Q65–Q79). As shown in Figure 3, congenital malformations and deformations of the musculoskeletal system (Q65–Q79) was the main type of the birth defects in central China livebirths, followed by cleft lip and cleft palate (Q35–Q37) and congenital malformations of eye, ear, face and neck (Q10–Q18). The types of congenital malformations of the respiratory system (Q30–Q34), other congenital malformations (Q80–Q89) and chromosomal abnormalities (Q90–Q99) were not observed. More detailed classification on birth defects was shown in Table 1. As shown in Table 1, the top 3 types of birth defects were polydactyly (Q69), congenital malformation of the foot (Q66) and syndactyly (Q70).

**Table 1:** The birth defects classified by ICD-10.

Code	Types of birth defects	Percentage	Rank
Congenital malformations of eye, ear, face and neck (Q10–Q18)			
Q16	Congenital malformation of the ear causing hearing impairment	5.00%	4
Q17	Other congenital malformations of the ear	4.44%	5
Q18	Other congenital malformations of the face and neck	0.56%	12
Cleft lip and cleft palate (Q35–Q37)			
Q35	Cleft palate	3.89%	6
Q36	Cleft lip	5.00%	4
Q37	Cleft lip and palate	2.22%	9
Congenital malformations and deformations of the musculoskeletal system (Q65–Q79)			
Q65	Congenital malformations of the hip	1.11%	11
Q66	Congenital malformation of the foot	11.11%	2
Q67	Congenital musculoskeletal malformations of the head, face, spine, and thorax	0.56%	12
Q68	Other congenital musculoskeletal malformations	2.22%	9
Q69	Polydactyly	41.11%	1
Q70	Syndactyly	9.44%	3
Q71	Congenital malformation of short upper limb	1.67%	10
Q72	Congenital malformation of short lower limb	0.56%	12
Q74	Other congenital malformations of limbs	0.56%	12
Q75	Other congenital malformations of skull and facial bones	0.56%	12
Other birth defects			
Q02	Microcephalus	0.56%	12
Q21	Congenital malformation of the cardiac septum	1.67%	10
Q39	Congenital malformation of the esophagus	0.56%	12
Q42	Congenital absence, atresia, and stenosis of large intestine	2.78%	8
Q54	Hypospadias	3.33%	7
Q55	Other congenital malformations of male reproductive organs	0.56%	12
Q61	Congenital renal cystic disease	0.56%	12

Infants with more than one defect category were included in each applicable major defect category.

### 3.2 Characteristics of birth defect

#### 3.2.1 Characteristics of livebirths with birth defects

As shown in Table 2, livebirths in the overall birth defect group and Q65–Q79 subgroup had significantly shorter body length than those in the healthy group. However, these subtle differences (0.3 and 0.4 cm, respectively) showed no practical clinical significance, because a difference of 0.3 cm/0.4 cm May not have much effect on the future heights of livebirths. Second, livebirths in the overall birth defect group and the three subgroups all had poorer health than those in the healthy group. Although there is an association between birth defects and health status, the closeness degree is not very strong. The Contingency coefficients were 0.038, 0.018, 0.013, and 0.015, respectively. Third, although livebirths with birth defects had an earlier delivery gestational age, birth defects did not lead to a higher percentage of preterm births (more than 28 weeks gestation age but less than 37 weeks). However, it is worth noting that the gestational age at delivery were 37.76 ± 1.6 week in the Q10–Q18 subgroup and 38.68 ± 1.412 week in the healthy group, and the difference was statistically significant. The 95% confidence interval (CI) for gestational age at delivery in the Q10–Q18 subgroup ranged from 36.16 to 39.36 week, implying that some livebirths in the Q10–Q18 subgroup were premature babies (<37 gestational weeks). The relationship between Q10–Q18 subgroup and gestational age at delivery May have clinical significance, which needs to be verified by studies with a larger sample size. Fourth, the birth defect group had a similar birth weight to the health group, but the birth defect groups had a higher proportion of babies with low birth weight (<2,500 g) (the Q10–Q18 subgroup was an exception), although the correlation degrees were not strong. Fifth, livebirths with birth defects were mainly male (63.9% vs. 36.1% in overall group and 62.9% vs. 37.1% in Q65–Q79 subgroup, *p* = 0.001 and 0.013). The association degrees between gender and overall birth defects and Q65–Q79 subgroup were 1.5 and 1.2%, respectively. Differences in constituent ratio of gender between Q35–Q37 and Q10–Q18 subgroups and healthy group were not observed. Sixth, compared with healthy newborns, birth defects did not result in significant differences in placenta position, malpresentation and mode of delivery. There was no increase in the prevalence of birth defects due to multiple births and test-tube baby.

**Table 2:** Characteristics of newborns with birth defects.

Variables	Health group (*n* = 43,402)	Birth defect group				p/C*			
		Overall (*n* = 166)	Q65–Q79 (*n* = 116)	Q35–Q37 (*n* = 20)	Q10–Q18 (*n* = 17)	Overall	Q65–Q79	Q35–Q37	Q10–Q18
Length of the baby (cm)	49.91 ± 1.50	49.61 ± 1.63	49.51 ± 1.83	49.75 ± 1.07	19.53 ± 1.07	**0.020**	**0.022**	0.642	0.301
Birth weight of the newborns (g)	3,246 ± 437	3,187 ± 488	3,177 ± 513	3,164 ± 509	3,064 ± 497	0.081	0.089	0.398	0.086
Gestational age at delivery (week)	38.68 ± 1.412	38.46 ± 1.43	38.47 ± 1.45	38.45 ± 1.46	37.76 ± 1.6	**0.046**	0.122	0.472	**0.008**
Premature infant						0.826	0.667	1.000	0.078
No	41,146(94.8%)	158(95.2%)	111(95.7%)	19(95%)	14(82.4%)				
Yes	2,256(5.2%)	8(4.8%)	5(4.3%)	1(5%)	3(17.6%)				
Low birth weight infant						**0.014/0.012**	**0.036/0.011**	**0.035/0.013**	0.256
No	41,816(96.3%)	154(92.8%)	107(92.2%)	17(85%)	15(88.2%)				
Yes	1,586(3.7%)	12(7.2%)	9(7.8%)	3(15%)	2(11.8%)				
Placenta previa						0.543	0.371	1.000	1.000
No	43,346(99.9%)	165(99.4%)	115(99.1%)	20(100%)	17(100%)				
Yes	56(0.1%)	1(0.6%)	1(0.9%)	0(0%)	0(0%)				
Breech or transverse position						0.787	0.479	0.633	0.479
No	42,913(98.9%)	165(99.4%)	116(100%)	20(100%)	17(100%)				
Yes	489(1.1%)	1(0.6%)	0(0%)	0(0%)	0(0%)				
Delivery mode						0.117	0.188	0.237	0.224
Vaginal delivery	16,421(37.8%)	53(31.9%)	79(68.1%)	15(75%)	13(76.5%)				
Cesarean section	26,981(62.2%)	113(68.1%)	37(31.9%)	5(25%)	4(23.5%)				
Gender						**0.001/0.015**	**0.013/0.012**	0.745	0.539
Male	22,296(51.4%)	106(63.9%)	73(62.9%)	11(55%)	10(58.8%)				
Female	21,106(48.6%)	60(36.1%)	43(37.1%)	9(45%)	7(41.2%)				
Health status of newborns						**<0.001/0.038**	**<0.001/0.018**	**0.024//0.013**	**0.012/0.015**
Good	39,693(91.5%)	123(74.1%)	95(81.9%)	15(75%)	12(70.6%)				
Average or poor	3,709(8.5%)	43(25.9%)	21(18.1%)	5(25%)	5(29.4%)				
Test-tube baby						0.796	0.990	0.628	1.000
No	42,847(98.7%)	163(98.2%)	114(2%)	19(95%)	17(100%)				
Yes	555(1.3%)	3(1.8%)	2(1.7%)	1(5%)	0(0%)				
Multiple pregnancy						1.000	0.674	0.380	1.000
No	43,066(99.2%)	165(99.4%)	116(100%)	19(95%)	17(100%)				
Yes	336(0.8%)	1(0.6%)	0(0%)	1(5%)	0(0%)				
Season of conception^†^						0.412	0.281	0.555	0.877
Spring	11,324(26.1%)	54(32.5%)	38(32.8%)	3(15%)	4(23.5%)				
Summer	10,419(24.0%)	37(22.3%)	29(25.0%)	7(35%)	3(17.6%)				
Autumn	9,928(22.9%)	33(19.9%)	25(21.6%)	4(20%)	5(29.4%)				
Winter	11,731(27.0%)	42(25.3%)	24(20.7%)	6(30%)	5(29.4%)				

The *p-*values were calculated using Two-sample *t-*test, Chi-square test, Fisher’s exact test or Mann–Whitney *U* test according to the type of variables. Variables with statistical significance were shown in boldface. ^†^Spring included March, April, and May. Summer included June, July, and August. Autumn included September, October, and November. Winter included December, January, and February. Q10–Q18: congenital malformations of eye, ear, face and neck; Q35–Q37: cleft lip and cleft palate; Q65–Q79: congenital malformations and deformations of the musculoskeletal system. *C*, contingency coefficient. *The value of *C* was shown when the Chi-square test showed that the difference in constituent ratio between the two groups was statistically significant and the correlation between the two variables exists.

#### 3.2.2 Characteristics of parents of livebirths with birth defects

As shown in Table 3, paternal ages of livebirths in overall birth defects, Q65–Q79 and Q35–Q37 subgroups were older than those in the healthy group, and the proportion of fathers older than 40 years was higher in the overall birth defects and Q65–Q79 subgroup than that in the health group (closeness degree: 1.7 and 1.6%, respectively), whereas there was no relationship between birth defects and paternal age in the Q10–Q18 subgroup. There was no significant difference in the composition ratio of race, occupation, education level, household registration address and nature between the health group and birth defect groups.

**Table 3:** Characteristics of fathers of newborns with birth defects.

Variables	Health group (*n* = 43,402)	Birth defect group				p/C*			
		Overall (*n* = 166)	Q65–Q79 (*n* = 116)	Q35–Q37 (*n* = 20)	Q10–Q18 (*n* = 17)	Overall	Q65–Q79	Q35–Q37	Q10–Q18
Age (year)	31.01 ± 4.92	32.56 ± 5.16	32.61 ± 5.06	33.72 ± 5.60	31.47 ± 3.94	**<0.001**	**0.001**	**0.019**	**0.718**
<40	37,832(87.2%)	139(83.7%)	98(84.5%)	16(80%)	14(82.4%)	**<0.001/0.017**	**0.002/0.016**	0.616	0.580
≥40	2,250(5.2%)	19(11.4%)	14(12.1%)	2(10%)	1(5.9%)				
Not stated	3,320(7.6%)	8(4.8%)	4(3.4%)	2(10%)	2(11.8%)				
Race									
Han	43,271(99.7%)	164(98.8%)	114(98.3%)	20(100%)	17(100%)	0.092	0.054	0.941	0.950
Minority	131(0.3%)	2(1.2%)	2(1.7%)	0(0%)	0(0%)				
Local inhabitant									
Yes	43,005(99.1%)	165(99.4%)	115(99.1%)	20(100%)	17(100%)	0.551	1.000	0.832	1.000
No	397(0.9%)	1(0.6%)	1(0.9%)	0(0%)	0(0%)				
The nature of household registration						0.280	0.488	0.490	1.000
Agriculture	41,966(96.7%)	163(98.2%)	114(98.3%)	19(95%)	17(100%)				
Non-agricultural	1,436(3.3%)	3(1.8%)	2(1.7%)	1(5%)	0(0%)				
Occupation						0.906	0.828	0.309	0.871
Staff of administrative, enterprise or institution	29,328(67.6%)	113(68.1%)	82(70.7%)	12(60%)	13(76.5%)				
Freelancer	5,727(13.2%)	23(13.9%)	14(12.1%)	5(25%)	2(11.8%)				
Other practitioners	8,347(19.2%)	30(18.1%)	17(14.7%)	3(15%)	2(11.8%)				
Education						0.873	0.863	0.735	1.000
Junior middle school or below	1,875(4.3%)	6(3.6%)	4(3.4%)	1(5%)	0(0%)				
High school or technical school	36,232(83.5%)	139(83.7%)	97(83.6%)	17(85%)	16(94.1%)				
College or above	895(2.1%)	2(1.2%)	1(0.9%)	0(0%)	0(0%)				
Not stated	4,400(10.1%)	19(11.4%)	14(12.1%)	2(10%)	1(5.9%)				

The *p-*values were calculated using Two-sample *t* test, Chi-square test, Fisher’s exact test or Mann–Whitney *U* test according to the type of variables. Variables with statistical significance were shown in boldface. Q10–Q18: congenital malformations of eye, ear, face and neck; Q35–Q37: cleft lip and cleft palate; Q65–Q79: congenital malformations and deformations of the musculoskeletal system. C, contingency coefficient. *The value of *C* was shown when the Chi-square test showed that the difference in constituent ratio between the two groups was statistically significant and the correlation between the two variables exists.

As shown in Table 4, mothers of livebirths with birth defect were significantly older than those of healthy newborns, and the proportion of the mothers aged 35 years or older was higher. However, the associations between maternal age and birth defect were observed only in the overall birth defect and Q65–Q79 subgroup with a small magnitude of 0.013 and 0.014. No significant difference in the composition ratio of race, occupation, education level, household registration address and nature between the two groups, as well as in the mean of height, were observed.

**Table 4:** Basic characteristics of mothers of newborns with birth defects.

Variables	Health group (*n* = 43,402)	Birth defect group				p/C*			
		Overall (*n* = 166)	Q65–Q79 (*n* = 116)	Q35–Q37 (*n* = 20)	Q10–Q18 (*n* = 17)	Overall	Q65–Q79	Q35–Q37	Q10–Q18
Age (year)	28.11 ± 4.48	29.32 ± 4.66	29.46 ± 4.73	28.95 ± 4.67	28.88 ± 4.63	**0.001**	**0.001**	0.401	0.476
<35 years old	39,543(91.1%)	141(84.9%)	97(83.6%)	17(85%)	16(94.1%)	**0.005/0.013**	**0.005/0.014**	0.416	1.000
≥35 years old	3,859(8.9%)	25(15.1%)	19(16.4%)	3(15%)	1(5.9%)				
Height (cm)	159.09 ± 5.94	160.05 ± 4.99	159.87 ± 5.22	158.80 ± 6.74	160.11 ± 5.69	0.084	0.240	0.851	0.605
Race						0.624	0.139	1.000	1.000
Han	42,922(98.9%)	163(98.2%)	113(97.4%)	20(100%)	17(100%)				
Minority	480(1.1%)	3(1.8%)	3(2.6%)	0(0%)	0(0%)				
Local inhabitant						0.388	0.910	0.064	0.052
Yes	36,123(83.2%)	134(80.7%)	97(83.6%)	13(65%)	11(64.7%)				
No	7,279(16.8%)	32(19.3%)	19(16.4%)	7(35%)	6(35.3%)				
The nature of household registration						0.974	0.621	1.000	0.626
Agriculture	38,990(89.8%)	148(89.2%)	103(88.8%)	18(90%)	17(100%)				
Non-agricultural	2,869(6.6%)	11(6.6%)	9(7.8%)	1(5%)	0(0%)				
Not stated	1,543(3.6%)	7(4.2%)	4(3.4%)	1(5%)	0(0%)				
Occupation						0.569	0.746	0.566	0.135
Staff of administrative, enterprise or institution	28,408(65.5%)	106(3.9%)	78(67.2%)	12(60%)	9(52.9%)				
Freelancer	7,079(16.3%)	32(19.3%)	20(17.2%)	5(25%)	6(35.3%)				
Other practitioners	7,915(18.2%)	28(16.9%)	18(15.5%)	3(15%)	2(11.8%)				
Education						0.652	0.487	0.598	0.886
Junior middle school or below	4,298(9.9%)	16(9.6%)	9(7.8%)	3(15%)	2(11.8%)				
High school or technical school	34,261(78.9%)	129(77.7%)	90(77.6%)	15(75%)	15(88.2%)				
College or above	2,150(5.0%)	9(5.4%)	8(6.9%)	1(5%)	0(0%)				
Not stated	2,693(6.2%)	12(7.2%)	9(7.8%)	1(5%)	0(0%)				

The *p-*values were calculated using Two-sample *t-*test, Chi-square test, Fisher’s exact test or Mann–Whitney *U* test according to the type of variables. Variables with statistical significance were shown in boldface. Q10–Q18: congenital malformations of eye, ear, face and neck; Q35–Q37: cleft lip and cleft palate; Q65–Q79: congenital malformations and deformations of the musculoskeletal system. C: contingency coefficient. *The value of *C* was shown when the Chi-square test showed that the difference in constituent ratio between the two groups was statistically significant and the correlation between the two variables exists.

As shown in Table 5, compared with mothers of healthy newborns, mothers of livebirths with birth defects gained weight more quickly during pregnancy based on a higher basal weight before pregnancy although no significant difference, so that they had a higher weight at delivery. Subgroup analysis showed that this phenomenon was mainly evident in the Q65–Q79 subgroup, while no statistical differences were observed in the other subgroups. The 95% CI of weight at delivery of mothers in the health group ranged from 58.19 kg to 76.55 kg, while it ranged from 60.27 kg to 82.73 kg in the Q65–Q79 subgroup’ mothers, indicating that some mothers in the Q65–Q79 subgroup reached a weight at delivery of more than 80 kg. As we mentioned above (2.3 Definition), a maternal weight of more than 80 kg is a clinically meaningful risk weight. Therefore, the association between maternal weight at delivery and birth defects of Q65–Q79 May be meaningful, but further studies with larger sample sizes are needed to verify it. In addition, mothers in overall birth defects group and Q65–Q79 subgroup had higher first-trimester fasting blood glucose. However, this minor difference May not be clinically significant, which did not result in fasting blood glucose values outside the normal reference range (3.9–6.1 mmol/L) in both the health group and the overall birth defects, as well as the subgroups. Moreover, mothers of birth defect group had higher gravidity and parity, and a higher proportion of exposure to suspected teratogens, high-risk pregnancies, and history of abnormal pregnancy-labor. However, Contingency coefficient showed that the associations of these variables with birth defects were not strong. There was no significant difference in blood pressure and menstruation. Among the mothers, 13.4% were underweight (BMI: <18.5 kg/m^2^), 66.2% were normal weight (BMI: 18.5–23.9 kg/m^2^), 18.5% were overweight (BMI: 24.0–29.9 kg/m^2^), and 1.9% were obese (BMI: ≥30 kg/m^2^) (not shown).

**Table 5:** Clinical characteristics of mothers of newborns with birth defects.

Variables	Health group (*n* = 43,402)	Birth defect group				p/C*			
		Overall (*n* = 166)	Q65–Q79 (*n* = 116)	Q35–Q37 (*n* = 20)	Q10–Q18 (*n* = 17)	Overall	Q65–Q79	Q35–Q37	Q10–Q18
Basal weight (kg)	55.31 ± 8.84	56.57 ± 10.78	57.12 ± 11.49	55.39 ± 10.88	57.26 ± 7.08	0.185	0.135	0.973	0.464
Weight at 24–28 weeks of gestation (kg)	61.74 ± 8.70	63.79 ± 10.42	64.68 ± 10.99	61.66 ± 8.57	61.74 ± 8.30	0.062	**0.032**	0.978	0.999
Weight at delivery (kg)	67.37 ± 9.18	70.70 ± 10.65	71.50 ± 11.23	69.87 ± 11.06	67.94 ± 7.60	**<0.001**	**0.001**	0.291	0.816
Weight gained (kg)	12.38 ± 5.24	13.53 ± 5.23	13.33 ± 5.10	15.33 ± 4.75	10.88 ± 5.99	**0.017**	0.098	**0.042**	0.343
Basal systolic blood pressure (mmHg)	108.26 ± 10.59	109.37 ± 10.16	109.7 ± 10.05	107.8 ± 10.76	113.1 ± 8.91	0.251	0.207	0.868	0.148
Basal Diastolic blood pressure (mmHg)	68.14 ± 8.23	68.86 ± 8.68	69.20 ± 8.67	67.27 ± 7.35	70.30 ± 7.50	0.343	0.239	0.680	0.408
Number of prenatal visits (time)	5.53 ± 2.83	5.83 ± 2.69	5.88 ± 2.65	5.93 ± 2.31	5.07 ± 3.05	0.220	0.235	0.584	0.543
First-trimester fasting blood glucose	4.68 ± 0.59	4.85 ± 0.58	4.94 ± 0.62	4.67 ± 0.40	4.60 ± 0.24	**0.012**	**0.001**	0.901	0.744
Gravidity (time)						**<0.001/0.019**	**<0.001/0.019**	0.127	**0.049/0.010**
1–2	27,696(63.8%)	86(51.8%)	81(69.8%)	14(70%)	11(64.7%)				
≥3	15,706(36.2%)	80(48.2%)	35(30.2%)	6(30%)	6(35.3%)				
Parity (time)						**0.025/0.012**	0.138	0.291	**0.003/0.024**
1–2	42,663(98.3%)	159(95.8%)	112(96.6%)	19(95%)	14(82.4%)				
≥3	739(1.7%)	7(4.2%)	4(3.4%)	1(5%)	3(17.6%)				
Age of menarche (year)	13.04 ± 0.65	12.95 ± 0.74	12.88 ± 0.71	13.20 ± 1.09	13.40 ± 0.55	0.373	0.219	0.585	0.217
Menstrual period (day)	5.31 ± 0.90	5.30 ± 0.79	5.40 ± 0.82	4.75 ± 0.50	6.00 ± 1.16	0.958	0.650	0.214	0.140
Menstrual cycle (day)	29.41 ± 2.27	29.23 ± 1.10	29.25 ± 1.16	28.50 ± 1.00	29.00 ± 1.16	0.675	0.757	0.424	0.720
Abnormal pregnancy-labor history						**0.041/0.010**	0.228	0.357	0.125
No	28,140(64.8%)	95(57.2%)	69(59.5%)	11(55%)	8(47.1%)				
Yes	15,262(35.2%)	71(42.8%)	47(40.5)	9(45%)	9(52.9%)				
History of miscarriage or induced labor						**0.004/0.014**	**0.042/0.010**	0.412	0.054
No	34,049(78.5%)	115(69.3%)	82(70.7%)	14(70%)	10(58.8%)				
Yes	9,353(21.5%)	51(30.7%)	34(29.3%)	6(30%)	7(41.2%)				
High-risk pregnancy						**0.001/0.016**	**<0.001/0.017**	0.432	0.691
No	27,546(63.5%)	84(50.6%)	55(47.4%)	11(55%)	10(58.8%)				
Yes	15,856(36.5%)	82(49.4%)	61(52.6%)	9(45%)	7(41.2%)				
Exposure to suspected teratogens in the first trimester of pregnancy						**0.032/0.011**	0.086	0.446	1.000
No	42,141(97.1%)	156(94.0)	109(94%)	19(95%)	17(100%)				
Yes	1,261(2.9%)	10(6.0%)	7(6%)	1(5%)	0(0%)				

The *p-*values were calculated using Two-sample *t-*test, Chi-square test or Fisher’s exact test. Variables with statistical significance were shown in boldface. Q10–Q18: congenital malformations of eye, ear, face and neck; Q35–Q37: cleft lip and cleft palate; Q65–Q79: congenital malformations and deformations of the musculoskeletal system. *C*, contingency coefficient. *The value of *C* was shown when the Chi-square test showed that the difference in constituent ratio between the two groups was statistically significant and the correlation between the two variables exists.

### 3.3 Risk factors for birth defects

**Figure 4:**
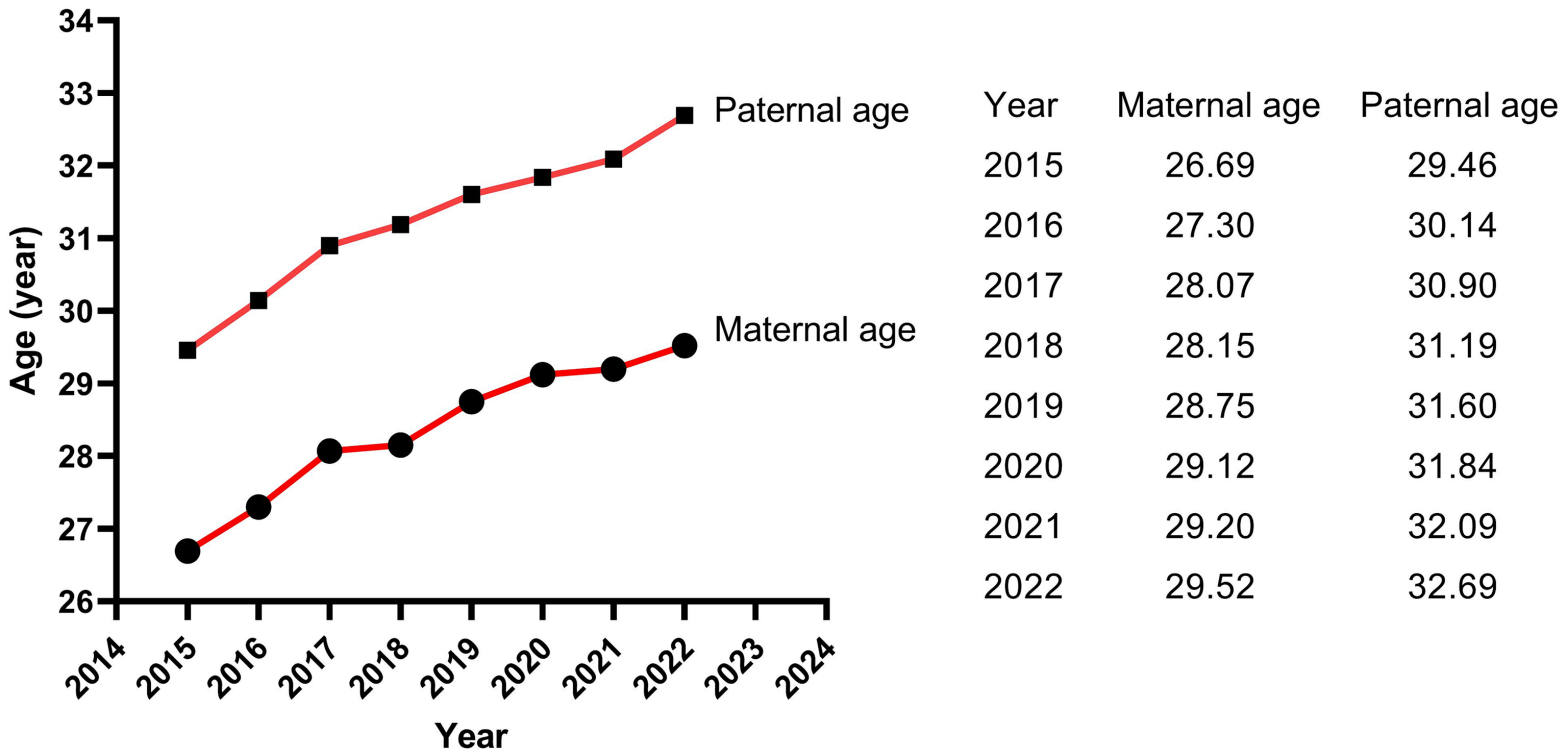
The trends of childbearing age of parents.

**Figure 5:**
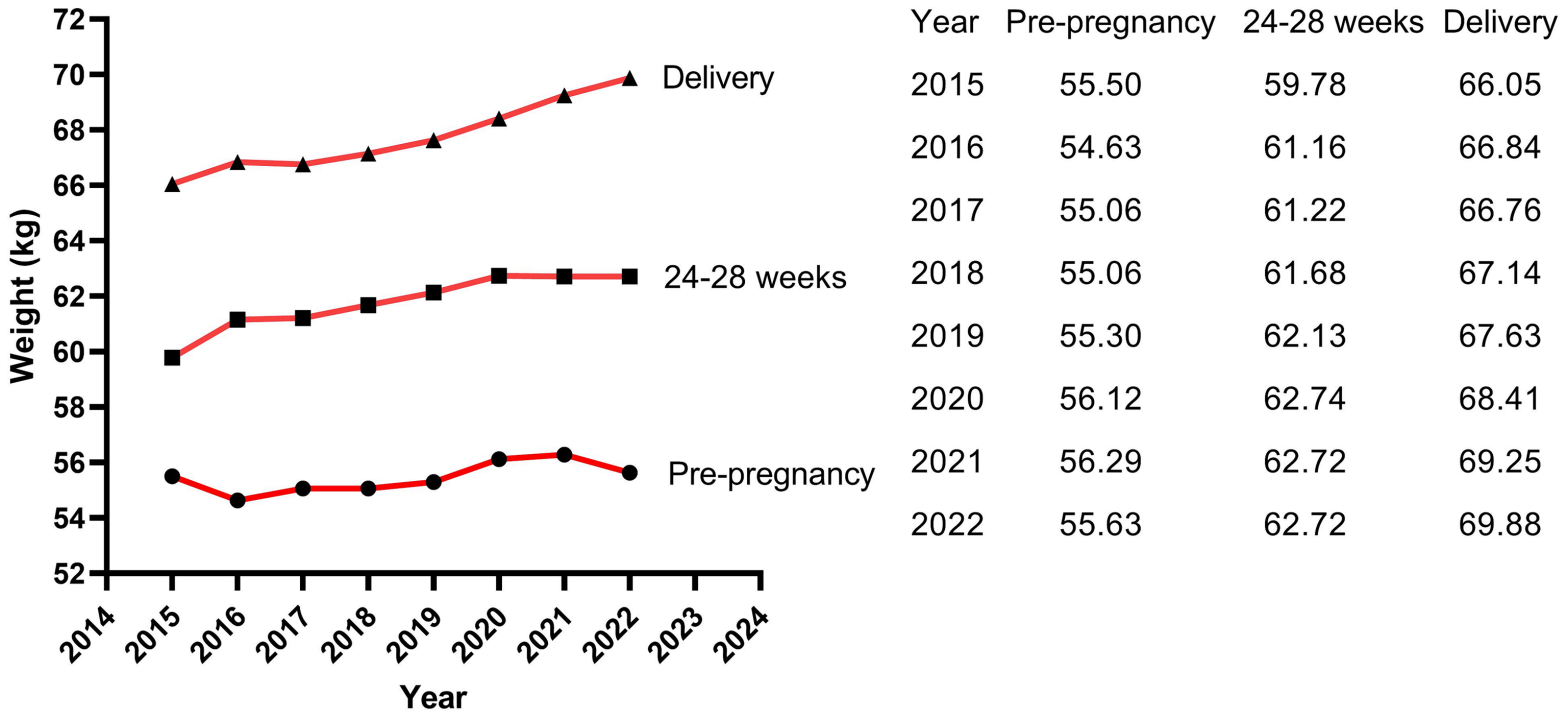
The trends of maternal weight.

The results of Poisson regression are shown in Tables 6–9. Overall, several risk factors are associated with birth defects, and the risk factors vary dramatically across specific defects. One risk factor May be associated with several defects, and one defect May be associated with several risk factors. Regression analysis showed that advanced paternal age (≥40 years) and excessive maternal weight at delivery (≥80 kg) were independent risk factors for overall birth defects. Gender of livebirths and excessive maternal weight at delivery (≥80 kg) and Gravidity ≥3times were independent risk factor for Q65–Q79. Among all the factors analyzed, Q35–Q37 was only affected by father’s age, and Q10–Q18 was only affected by parity ≥3 times. Moreover, as the calendar year increases, the risk of birth defects increases. Given that paternal age and maternal weight were independent risk factors for overall birth defects, we further analyzed trends of these two factors from 2015 to 2022, the results were shown in Figures 4, 5. The results showed that both the age of parents and maternal weight at delivery showed an upward trend.

**Table 6:** Risk factor for overall birth defects.

Variables	Univariate analysis		Multivariate analysis	
	Crude PRR (95%CI)	*p*	Adjusted PRR (95%CI)	*p*
Fetus				
Gender				
Female	1.00		1.00	
Male	1.669(1.216, 2.291)	**0.002**	1.400(0.882,2.223)	0.154
**Father**				
Age (year)				
<40	1.00		1.00	
≥40	2.287(1.416, 3.694)	**0.001**	2.532(1.201,5.336)	**0.015**
Mother				
Age (year)				
<35	1.00		1.00	
≥35	1.812(1.184, 2.772)	**0.006**	0.661(0.298,1.466)	0.308
Weight at delivery (kg)				
<80	1.00		1.00	
≥80	2.170(1.404, 3.352)	**<0.001**	1.785(1.006,3.166)	**0.048**
First-trimester fasting blood glucose	1.418(1.098, 1.832)	**0.008**	1.203(0.860,1.683)	0.281
Gravidity (time)				
1–2 times	1.00		1.00	
≥3times	1.980(1.412, 2.775)	**<0.001**	1.623(0.907,2.904)	0.103
Parity (time)				
1–2 times	1.00		1.00	
≥3 times	2.527(1.185, 5.387)	**0.016**	0.749(0.177,3.167)	0.694
History of abnormal pregnancy-labor				
No	1.00		1.00	
Yes	1.376(1.012, 1.872)	**0.042**	0.757(0.371,1.545)	0.444
History of miscarriage or induced labor				
No	1.00		1.00	
Yes	1.611(1.159, 2.240)	**0.005**	1.182(0.606,2.307)	0.623
High-risk pregnancy				
No	1.00		1.00	
Yes	1.692(1.248, 2.294)	**0.001**	1.604(0.854,3.013)	0.142
Exposure to suspected teratogens in the first trimester of pregnancy				
No	1.00		1.00	
Yes	2.133(1.126, 4.043)	**0.020**	1.670(0.745,3.741)	0.213
Calendar year	1.406(1.298, 1.523)	**<0.001**	1.399(1.215, 1.610)	**<0.001**

Variables with statistical significance were shown in boldface. CI, confidence intervals; PRR, prevalence rate ratio.

**Table 7:** Risk factor for Q65–Q79.

Variables	Univariate analysis		Multivariate analysis	
	Crude PRR (95%CI)	*p*	Adjusted PRR (95%CI)	*p*
Fetus				
Gender				
Female	1.00		1.00	
Male	1.605(1.101, 2.340)	**0.014**	1.624(1.059, 2.490)	**0.026**
Father				
Age (year)				
<40	1.00		1.00	
≥40	2.393(1.367, 4.190)	**0.002**	1.774(0.856, 3.679)	0.123
Mother				
Age (year)				
<35	1.00		1.00	
≥35	2.002(1.224, 3.274)	**0.006**	1.049(0.521,2.109)	0.894
Weight at delivery (kg)				
<80	1.00		1.00	
≥80	2.408(1.455, 3.987)	**0.001**	2.153(1.282, 3.614)	**0.004**
Gravidity (time)				
1–2 times	1.00		1.00	
≥3times	2.167(1.458, 3.222)	**<0.001**	2.062(1.238, 3.433)	**0.005**
History of miscarriage or induced labor				
No	1.00		1.00	
Yes	1.508(1.011, 2.249)	**0.044**	1.013(0.616,1.667)	0.959
High-risk pregnancy				
No	1.00		1.00	
Yes	1.923(1.336, 2.769)	**0.001**	1.239(0.765,2.006)	0.385

Variables with statistical significance were shown in boldface. Q65–Q79: congenital malformations and deformations of the musculoskeletal system. CI, confidence intervals; PRR, prevalence rate ratio.

**Table 8:** Risk factor for Q35–Q37.

Variables	Univariate analysis		Multivariate analysis	
	Crude PRR (95%CI)	*p*	Adjusted PRR (95%CI)	*p*
Father				
Age (year)	1.100(1.016,1.191)	**0.019**	1.141(1.045,1.258)	**0.004**
Mother				
Weight gained (kg)	1.104(1.004,1.214)	**0.040**	1.090(0.987,1.204)	0.088

Variables with statistical significance were shown in boldface. Q35–Q37: cleft lip and cleft palate. CI, confidence intervals; PRR, prevalence rate ratio.

**Table 9:** Risk factor for Q10–Q18.

Variables	Univariate analysis		Multivariate analysis	
	Crude PRR (95%CI)	*p*	Adjusted PRR (95%CI)	*p*
Mother				
Gravidity (time)				
1–2 times	1.00		1.00	
≥3 times	2.742(1.014, 7.413)	**0.047**	1.822(0.597,5.565)	0.292
Parity (time)				
1–2 times	1.00		1.00	
≥3 times	12.325(3.542, 42.887)	**<0.001**	8.751(2.159, 35.478)	**0.002**

Variables with statistical significance were shown in boldface. Q10–Q18: congenital malformations of eye, ear, face and neck. CI, confidence intervals; PRR, prevalence rate ratio.

## 4 Discussion

Hubei Province is located in the central region of China, with well-developed transportation and relatively well medical condition in China. Our study showed that the overall livebirths prevalence of birth defects from 2015 to 2022 in Jingzhou city, Hubei Province, China, is 3.81per 1,000 livebirths, which is lower than the prevalence of other areas of China. The prevalence of birth defects in Hunan Province, which borders Hubei Province, was 19.18 per 1,000 perinatal infants from 2005 to 2014 (4) and 22.05 per 1,000 perinatal infants from 2014 to 2018 (8). The prevalence of birth defects in Zhejiang Provinces located in eastern China was 18.32 per 1,000 births (9). The prevalence of birth defects in Guangxi Province located in southwest China was 12.17 per 1,000 perinatal infants (15). In addition, the prevalence rate in Hubei Province was obviously lower than those of other ethnic populations in other countries, such as Korean (28.69 per 1,000 livebirths, 2005–2006) (5), Europe (23.9 per 1,000 births, 2003–2007) (16) and Africa (23.5 per 1,000 newborns, 2023) (17). However, the prevalence in Hubei Province was similar to that in Jiangsu Province which was 7.15 per 1,000 perinatal infants (3). Similarly, the prevalence in our study population was lower than that of newborns born to Taiwan native-born mothers (28.6 per 1,000 newborns), but similar to newborns born to immigrants from mainland China (9.8 per 1,000 newborns) (14). Another study from Hubei Province also showed that the prevalence of birth defects which was 8 per 1,000 livebirths in this region was significantly lower than that in other regions, ranking in the moderate to lower level of China (18). In this study, the reasons for the low prevalence of birth defects in Hubei Province are mainly considered as follows: (1) the birth defects in our study were all diagnosed within 28 weeks of gestation to 7 days after birth, excluding fetuses aborted, induced and stillborn due to birth defects and newborns with birth defects diagnosed after 7 days after birth; (2) China implemented a universal two-child policy in 2016. The prevalence of birth defects was relatively low due to the relatively younger age of mothers and fewer gravidity and parity before the two-child policy, and, after the two-child policy implementation, the prevalence of birth defects increased significantly (9). Our study included data of newborns born in 2015 and 2016, which contributed to the overall low prevalence in our study; (3) Most of our subjects were from rural areas, where the prevalence of birth defects is lower than that in urban areas (14).

However, it is worth noting that the prevalence of our study in 2022 also reached 9.23 per 1,000 livebirths, showing a drastically uptrend. Moreover, consistent with the results of Lin et al. (14), the main type of birth defect in Hubei Province is congenital malformations and deformations of the musculoskeletal system (Q65–Q79) accounted for 68.4% and cleft lip and cleft palate (Q35–Q37) accounted for 11.7%, which were not fatal defects. In contrast, congenital heart disease, neural tube defects and Down syndrome, which were more common before (9, 15), showed small proportions in our study population. This May be attributed to the relatively advanced prenatal screening and diagnostic technology and policy supports in Hubei Province. This May be also one of the reasons why the livebirths prevalence of birth defects in Hubei Province is lower than those in other regions, because most fetuses with those serious birth defects were induced after prenatal diagnosis, which were excluded from the statistical analysis of this study. Moreover, this study included only livebirths birth defects detected within the first 7 days of life, which May contribute to the low prevalence of congenital heart disease showed in this study.

Moreover, our findings showed that January and February were the peak months for livebirths birth defects, although we did not identify significant difference in the prevalence rates of birth defects in different seasons of conception. Zhou et al. (3) reported the consistent results as ours in Jiangsu, China, while studies in other countries and regions have shown seasonal variations in birth defects (19–22). Benavides et al. (22) suggested that season of conception was associated with 5% of birth defects in Texas and summer conception was associated with any monitored birth defect and five specific phenotypes, most notably Hirschsprung disease. de la Vega and López-Cepero (19) detected a statistically significant increase in the incidence and relative risk during the summer months (using winter as a reference) of conceiving a child with open neural tube defects, cardiac anomalies, or cleft lip and palate in Puerto Rico. Possible reasons for this lack of consistency in findings include differences in populations, underlying factors, seasons or climates, diet and lifestyle, and methods of analysis between the studies. In our study, the majority of newborns delivered in January and February were conceived in April and May in spring. The highest rate of birth defects in our study was 32.5% in spring conception and the lowest was 19.9% in autumn conception. Women who were pregnant in spring were immediately subjected to summer and autumn when watermelon, lychee, durian and other high-sugar melons and fruits were ripe after the early pregnancy reaction, while women who were pregnant in autumn were followed by winter and spring when there were fewer high-sugar melons and fruits after the early pregnancy reaction. As a result, women who conceive in the spring were more likely to develop gestational diabetes mellitus and rapid weight gain, as well as other pregnancy complications that came with them. Diabetes and excess weight have both been reported to increase the risk of birth defects (11, 14, 23–26).

Our study showed that livebirths with specific birth defects had poorer health, earlier delivery gestational age, and were more likely to suffer from low body weight, which might carry medical, surgical, cosmetic, or lifestyle consequences (27). Moreover, perinatal infants with Q65–Q79 birth defects were predominantly male (62.9% vs. 37.1%, *p* = 0.001) and male newborns had a 1.624 times risk of birth defects than female newborns. This phenomenon has been verified in many studies (3, 4, 8). This might be due to several reasons. (1) Recessive defects on the father’s X chromosome are more likely to show up in boys. (2) Y chromosome has a higher susceptibility than X chromosome (28). (3) Female fetuses with birth defects are easier to be induced, while male fetuses with minor birth defects are more likely to be retained due to China’s traditional preference for sons. (4) The external genital deformities in males are more detectable than in females (4).

Previous studies have commonly studied the relationship between maternal factors and birth defects, while few studies on paternal factors. In this study, we also collected data on fathers of perinatal infants to analyze the influence of paternal factors on birth defects. Our study showed that the paternal ages of livebirths in overall birth defects, Q65–Q79 and Q35–Q37 subgroup were significantly higher than that in healthy group, and advanced paternal age (≥40 years) was an independent risk factor for overall birth defects and Q35–Q37. The association could be caused by mutations of the gametes in men induced by biological or environmental factors, because spontaneous mutations in germ cells increase with male age (29). Bu et al. (30) suggested that advanced paternal age > 44 years was associated with increased risk of congenital anomalies after adjusting confounding factors in the USA, mainly for the chromosomal anomalies, but not the structure anomalies. Gili et al. (31) believed that advanced paternal age was a risk factor for preaxial polydactyly in South American. A meta-analysis of Fang et al. (32) indicated that paternal age is associated with a moderate increase in the incidence of urogenital and cardiovascular abnormalities, facial deformities, and chromosome disorders. However, study of Hurley and DeFranco (33) did not find a correlation between paternal age and birth defects in Ohio of American. Similar, no differences in paternal age were observed between cases and controls in study of Nazer et al. (34). Thus, the influence of advanced paternal age on birth defects is related to race and region. Men in Hubei Province, China, should be advised to have children at the appropriate age, and expectant fathers with advanced age should pay more attention to prenatal screening and diagnosis. As a risk-based recommendation, Friedman (35) suggests that men should complete their families before age 40.

For mother’s factors, advanced age, rapid weight gain, excess weight at delivery, high first-trimester fasting blood glucose, more than 2 times of gravidity, more than 2 times of parity, abnormal pregnancy-labor history, history of miscarriage or induced labor, high-risk pregnancy and exposure to suspected teratogens in the first trimester of pregnancy all increased the risk of overall birth defects, while only excess weight at delivery (≥80 kg) was independent risk factors for birth defects (especially for Q65–Q79). Previous studies focused on mother’s pre-pregnancy weight (36–48), and few studies have paid attention to weight gain and weight at delivery. Obviously, in our study population, excess weight at delivery was the stronger factor than mother’s pre-pregnancy weight and weight gain during pregnancy. Previous studies have shown that pre-pregnancy overweight or obesity was associated with the increased risk of birth defects in population of Texas (39), Florida (40) and so on. However, this association was not significant in our study population. We considered that the limited sample size, low proportion of overweight (18.5%) and obese (1.9%) pregnant woman, and low prevalence of birth defects might lead to the insufficient sensitivity to this association in our study. It was reported that the percentage of overweight and obese pregnant woman was 67% in Texas (39) and 42.5% in Florida (40). Therefore, a larger sample size study is more suited to verify the relationship between pre-pregnancy weight and birth defects in Hubei Province of China in the future. In our study, the weight of mothers in the birth defect group was similar to that in the healthy group at pre-pregnancy (56.57 kg vs. Fifty five 0.31 kg, *p* = 0.185), showed borderline significance at 24–28 weeks of gestation (63.79 kg vs. Sixty one 0.74 kg, *p* = 0.062), and reached statistical significance at delivery (70.70 kg vs. Sixty seven 0.37 kg, *p* < 0.001), indicating that mothers in the birth defect group gained more weight during pregnancy than those in the healthy group. The difference of weight gained also showed statistical significance between the two groups in our study. However, variable of weight gained did not remain significant in multivariate regression, suggesting that weight gained was not as strongly associated with birth defects as weight at delivery. However, to our knowledge, this was the first study to analyze the relationship between weight at delivery and birth defects, so the mechanisms behind this association were unclear. The possible reason May be due to the fact that the weight at delivery was a result of continuous accumulation of pregnancy weight gained on the basis of pre-pregnancy weight, which reflected status of both pre-pregnancy weight and pregnancy weight gained. Pre-pregnancy overweight or obesity has been shown to increase the risk of birth defects, while rapid weight gain during pregnancy led to an increased risk of gestational diabetes mellitus (GDM) (41) which has been reported to increase the risk of birth defects (26). Of course, since GDM was typically diagnosed in the second trimester (24–28 gestational weeks), GDM should not be considered as a direct risk factor for birth defects but rather as a signal of a longer-term metabolic dysfunction of the mother, which affects at the time of meiosis and early pregnancy (26). Moreover, it was reported that obese pregnant women have increased inflammation and oxidative stress, and lower levels of nutritional antioxidant defenses compared with lean pregnant women, which may contribute to the adverse outcomes (42, 43). It was also reported that obese pregnant women transferred less 25(OH)D (44, 45) and iron (46) to their fetuses. In addition, pregnant women with exceeding weight at delivery tended to complicate pre-pregnancy diabetes or GDM, as well as many other pregnancy complications, which could increase the risk of birth defects (24, 47). Women with risk weight at delivery (80 kg) had either pre-pregnancy overweight or obesity, excessive gestational weight gain, or both. Women who had a higher pre-pregnancy weight and gained weight faster during pregnancy were more likely to subject to risk weight at delivery. The significance of pre-pregnancy overweight or obesity for pregnancy care May be limited, while simultaneously controlling pre-pregnancy weight and gestational weight gain to reduce weight at delivery May be more meaningful for the prevention of birth defects in Hubei Province where has a low proportion of obese pregnant women. In conclusion, weight control before pregnancy and physical exercise during pregnancy are crucial for obese pregnant women to control their weight at delivery and reduce the prevalence of birth defects (48).

Moreover, more than 2 times of gravidity was associated with an increased risk for Q65–Q79. The reasons are mainly considered as follows: (1) parents with more than 2 times of gravidity had an older age than those with gravidity≤2 times (34.61 years vs. Thirty 0.29 years for fathers and 31.60 years vs. Twenty seven 0.42 years for mothers in our study, all *p* < 0.001, not shown), and advanced age, especially advanced paternal age, was a risk factor for birth defects as we discussed above; (2) mothers with more than 2 times of gravidity managed their weight relatively poorly as the increase of their age (56.61 kg vs. Fifty five 0.05 kg for basal weight and 68.23 kg vs. Sixty seven 0.20 kg for weight at delivery in our study, all *p* < 0.001, not shown), which was a high-risk factor as we discussed above; (3) although China implemented a universal two-child policy in 2016 and three-child policy in 2021, the rate of newborn with more than 2 times of parity was only 1.7% (746/42822) in our study population. In other words, more mothers with more than 2 times of gravidity (16.6%, 7246/36322) in our study population usually had a history of miscarriage, induced labor or stillbirth, which were risk factors for birth defects (49). History of miscarriage or induced labor was not an independent risk factor for birth defects in our study because the data of birth defects on miscarriage, induced labor, and stillbirth was not included in our study. In addition, birth defects data from population-based birth defects surveillance system in China’s Jiangsu of Zhou et al. (50) also showed that gravidity ≥3 (PRR = 1.38) was risk factor for birth defects. Li et al. (51) reported that gravidity was associated with occurrence of congenital heart defects. Study of Zhang et al. (52) showed that the birth defect group had significantly higher gravidity than the control group. In addition, more than 2 times of parity was associated with an increased risk for Q10–Q79. The reason May be similar to excess gravidity. Therefore, mothers with more than 2 times of gravidity or parity should strengthen prenatal screening. However, pregnant women with risk factors mentioned above should all pay more attention to prenatal screening and diagnosis.

The reason that paternal age was an independent risk factor for birth defects while maternal age had a relatively small effect on birth defects May be partly attribute to the older mean childbearing age of fathers than that of mothers (31.02 ± 4.91 vs. 28.11 ± 4.48). As early as 1955, Penrose (53) demonstrated that the statistical association between parental ages and birth defects caused by fresh dominant mutations was largely attributable to the age of the father, not to mother’s age. Study of Lian and Zack (54) similarly indicated that older fathers had a higher risk for birth defects, while an equivalent association for older mothers was not found. These results indicated that results were flawed to some extent when risk factors were analyzed considering only the age of the mother.

It is worth noting that the livebirths prevalence of birth defects in our study showed a remarkable uptrend. Moreover, as the calendar year increased, the risk of birth defects for livebirths increased by 41.5% for each additional year. Study of Zhao et al. (2) based on the GBD2019 dataset showed that the variation trend of incidence for birth defects between 1990 and 2019 globally was not statistically significant, but showed an increasing trend in China (*p* < 0.001), with an annually percentage changes of 0.26%, and the upward trend was projected to continue between 2020 and 2030. The similar dramatic upward trend was also observed in China’s Jiangsu province (3), Guangxi province (7, 15), Hunan province (4) and Zhejiang province (9). Regarding etiology, although in many cases the intrinsic cause is still unexplicit, it has been hypothesized that birth defects May be caused by complex interactions between genes and environment, which modify the normal embryo-fetal development, especially during the organogenesis phase (55). The increasing risk and prevalence of birth defects May, to a large extent, attribute to deteriorating living and working environment, changes in lifestyle and habits, and changed conception on family planning. The air and drinking water quality deteriorated because of the rapid development of industry and the popularization of cars. It was reported that nitrate, agrichemicals and chlorination in drinking water during pregnancy increased the risk of birth defects (56–58). Several studies suggested that exposure to common air pollutants (SO_2_, PM_2.5_, PM_10_, NO_2_, O_3_, and CO) increased the risk of birth defects (59), with the maximum effect in the 7th or 8th week for PM_2.5_, the 7th week for SO_2_, the 8th week for PM_10_, the 7th week for NO_2_, and the 31st or 32nd week for O_3_ (60). Moreover, the fast pace of work and life, the increasing pressure from study and work, the convenient transportation and the online shopping, work and communication all contributed to fewer opportunities to exercise and weight gained (61, 62). With the enrichment of material life, a variety of sugar-sweetened beverages and foods and meat diet also led to obesity (63, 64). Obviously, obesity increases the risk of birth defects as shown in our study, and the weight at delivery of the mothers in our study increased from 66.04 kg in 2015 to 69.88 kg in 2022. In addition, the average childbearing age for mothers in our study increased from 26.69 years in 2015 to 29.52 years in 2022, and for fathers from 29.46 years in 2015 to 32.69 years in 2022. The childbearing age of the parents, especially the age of fathers, was a crucial factor affecting birth defects as shown in our study. On the other hand, the continuous advancement of medical level led to the induction of serious birth defects, the Decreased rate of stillbirth and the increased prevalence of birth defects in live births (2). Since birth defect represents a significant public health issue, an effective primary prevention strategy should be a priority for public policies and healthcare system.

Although we collected the detailed data for analysis, our study also had some shortcomings. First, our sample size was not very large, which limited the explore for risk factors, trend, seasonality of specific birth defect. Second, our study did not include data on miscarriage, induced labor and birth defects diagnosed after 7 days of birth, while most of the fetuses aborted or induced were due to birth defects, and many birth defects were not detected during 7 days after delivery. Third, although we collected fasting blood glucose values at the first trimester, information on pregestational diabetes and gestational diabetes was not collected due to the data incompleteness, though both of which have been reported to be associated with birth defects. Fourth, we did not analyze the relationship between maternal and paternal smoking and alcohol consumption and birth defects, because we found that many subjects hid these histories when we collect and collate the relevant data. Fifth, the study sample was from only one county which was not a representative sample, and the results cannot be generalized. Sixth, exposure to certain heavy metals like lead and arsenic, as well as the socio-economic status of the participant which might be risk factors for birth defects were not included into our study because they were not recorded in the database.

## 5 Conclusion

The livebirths prevalence and risk of birth defects in a county of Hubei Province of China showed a remarkable uptrend and elevated paternal age, excess maternal weight, gravidity or parity might be added to the array of factors that prospective parents consider when planning their families which warrants further investigations.

## Data Availability

The raw data supporting the conclusions of this article will be made available by the authors, without undue reservation.
